# Understanding Predictors of Lifelong Initiation and Follow-up Treatment for adolescents and youth living with HIV (UPLIFT): an integrated prospective cohort in Eastern Cape, South Africa

**DOI:** 10.1136/bmjopen-2024-092909

**Published:** 2025-07-08

**Authors:** Elona Toska, Olanrewaju Edun, Siyanai Zhou, Zea Leon, Nontokozo Langwenya, Janina Jochim, Janke Tolmay, Gayle Sherman, Lucie Dale Cluver

**Affiliations:** 1 Centre for Social Science Research, University of Cape Town Faculty of Humanities, Rondebosch, South Africa; 2 Department of Social Policy and Intervention, University of Oxford, Oxford, UK; 3 Imperial College London, London, UK; 4 Centre for Social Science Research University of Cape Town, Cape Town, South Africa; 5 University of the Witwatersrand, Johannesburg-Braamfontein, South Africa; 6 Department of Child and Adolescent Psychiatry and Mental Health, University of Cape Town, Cape Town, South Africa

**Keywords:** Adolescent, HIV & AIDS, Public health, SOCIAL MEDICINE, Africa South of the Sahara, Treatment Outcome

## Abstract

**Abstract:**

**Purpose:**

Adolescents living with HIV (ALHIV) are a priority population for achieving global HIV prevention and treatment targets but experience poorer outcomes than adults. Long-term follow-up is essential to understand their transition into adulthood. By linking self-reported survey data with routine laboratory records, we established a social science clinical cohort of ALHIV South Africa’s Eastern Cape to explore factors shaping their long-term health and well-being.

**Participants:**

Eligible participants were adolescents who were part of a three-wave quantitative cohort of ALHIV and not living with HIV (2014–2018) and had consented (adolescent and caregiver) to having their self-reported interviews linked with routine health records (n=1563). Adolescents were recruited into the existing three-wave cohort through clinic and community-based methods (97% enrolment, >90% retention over three waves). Between 2019 and 2022, we abstracted laboratory test records from the National Health Laboratory Services database for all eligible participants, with matching based on demographic variables. Individuals with at least one HIV-related record form our ‘lifelong social science cohort’, a total of 956 ALHIV (852 of 1107 ALHIV and 104 of 456 HIV-uninfected).

**Findings to date:**

A total of 32 886 laboratory test records from 2004 to 2023 were matched through three rounds of data extraction, using iteratively refined record-linking searches. Most records were viral load (8864) and CD4 count (6801) results, with a median of 10 (IQR: 7–14) and 8 (IQR: 5–11) tests per matched adolescent, respectively. Overall, 956 of 1563 adolescents (61%) were successfully linked to laboratory data, including 852 of 1107 (77%) ALHIV. Analysis of the matched cohort survey-laboratory data provided several insights. Self-reported antiretroviral therapy adherence was strongly associated with viral suppression, even after adjusting for covariates. The strongest predictors of suppression were not reporting missed doses in the past 3 days, past week and not missing clinic appointments in the past year. Among adolescent girls and young women living with HIV, access to safe and affordable facilities, and kind and respectful staff were associated with a higher likelihood of multiple improved HIV-related outcomes, including viral suppression. Exposure to sexual and intimate partner violence predicted worse viral load outcomes among adolescents.

**Future plans:**

This integrated prospective cohort provides an opportunity to characterise long-term HIV treatment outcomes among ALHIV in Africa. We will investigate how individual, familial, community and healthcare experiences in childhood, and adolescence shape these outcomes. Since the COVID-19 pandemic happened during the period of matched data, we will also investigate the potential effect of the COVID-19 pandemic on adolescent HIV treatment outcomes, with potential subgroup analyses for individuals with available COVID-19-related results.

**STRENGTHS AND LIMITATIONS OF THIS STUDY**

- The Understanding Predictors of Lifelong Initiation and Follow-up Treatment for adolescents living with HIV (ALHIV) study successfully linked data for a large cohort of ALHIV in South Africa from three rounds of interviews to routine National Health Laboratory Services (NHLS) laboratory test results. Interviews assessed HIV medication-related experiences, sexual and reproductive health practices, mental health well-being and social and structural factors.
- The NHLS is the preferred provider of laboratory tests conducted in public sector health facilities in South Africa including HIV tests for the national rollout of antiretroviral therapy since 2004. NHLS test records are stored digitally.
- This matched dataset includes routine laboratory tests conducted on adolescents who transfer out of the survey catchment area, enabling long-term outcome monitoring of HIV and health outcomes for young populations who may be mobile and accessing care in multiple facilities.
- The integrated prospective cohort provides a unique opportunity to understand how experiences during childhood and adolescence influence long-term immunologic and virologic patterns and HIV treatment outcomes among ALHIV, including HIV-related comorbidities and the effects of the COVID-19 pandemic.
- The inability to link 23% of eligible ALHIV to any test record in the NHLS, whether due to failed linkage or lack of testing, may limit the generalisability of findings based on these data.

## Introduction

 Improved survival of children with vertically acquired HIV on antiretroviral therapy (ART) and a high number of new adolescent HIV acquisitions, annually,^1^ have contributed to a growing cohort of adolescents (aged 10–19 years) living with HIV. In 2022 alone, there were an estimated 140 000 new HIV acquisitions among adolescents, with the highest proportion living in sub-Saharan Africa.^1^ Due to multiple biological, psychological, social and structural factors, adolescents living with HIV (ALHIV) have poor rates of adherence to ART, retention on treatment and viral suppression compared with adults.^2 3^ Understanding what factors can improve treatment outcomes in this population remains an important research priority given the paucity of evidence on effective interventions to improve adherence, retention and viral suppression.^4^,^7^ Importantly, understanding how the effect of these factors change over time as ALHIV transition from childhood to young adulthood is critical to maximising their survival and reaching human potential.

The ‘Mzantsi Wakho’ Adolescent Research study—a longitudinal cohort of ALHIV in Eastern Cape, South Africa—included three rounds of study interviews conducted between 2014 and 2018. The study aimed to investigate the individual, familial, social and structural factors associated with HIV and sexual and reproductive health (SRH) outcomes among ALHIV. Analyses of Mzantsi Wakho self-reported survey data have provided important evidence on how factors such as disclosure of HIV status, mental health symptoms, social and family relationships among adolescents influence multiple health outcomes, including ART adherence.^8^,^13^ After the first round of interviews (2015–2016), the research team conducted facility-based abstraction of clinical and laboratory data from participant clinic files to further understand how survey-reported factors influence health outcomes (mortality, TB infection, CD4 count and viral load (VL) levels),^14^ with individual adolescent and caregiver consent. Paper-based patient files, enhanced by electronic records from the Tier.net database system in hospitals for 88.1% (N=951) of ALHIV, were extracted from 52 health facilities in the catchment area in 2014–2016, prior to the national rollout of Tier.net to all facilities.^14^ While paper-based records were located for 90% of the participants, data abstracted from 75% of participants with any HIV-related records did not include any recent laboratory data (VL or CD4 count measurements): only 13% had any VL tests conducted in the 2 years prior to data abstraction. Reasons for poor data availability include logistical challenges (transportation of specimen, specimen quality, etc), poor data capturing into patient files and patient mobility between clinics.^14^ Moreover, the natural time effect of the abstraction process created incomparable periods between participants. Thus, innovative approaches that are less resource intensive but accommodate the mobile nature of adolescents and young people were needed to provide more exhaustive routine data to investigate the long-term HIV and health outcomes of this cohort.

Access to longitudinal clinic data among children and ALHIV has been useful for monitoring how HIV testing, treatment, retention and viral suppression patterns have evolved throughout the epidemic.^15^,^18^ Their use in South Africa’s national HIV programme^19^ and in cohorts such as the International Epidemiology Databases to Evaluate AIDS, an international research consortium, which collects observational data from clinical centres and research groups,^18 20^ have been critical in understanding the epidemiology of ALHIV in sub-Saharan Africa.^18 19 21^ However, the use of routine clinical data alone can be limited by the absence of data on adolescents’ experiences in their ecological environment, including disclosure of HIV status, mode of acquisition of HIV and family and social relationships status, which may influence their health outcomes. The linkage of laboratory data to social science survey data among ALHIV can improve the utility of both data sources. As ALHIV grow older and experience other unique challenges such as transition to adult care, access to their longitudinal clinical data provides the opportunity to assess how their long-term health outcomes evolve over time and identify what childhood or adolescent experience influence their future treatment trajectories.^22^

Linkage of data from cohort studies of people living with HIV to central laboratory databases has been used among adults to limit the challenge of data missingness often associated with using patient files,^23^ but not among ALHIV. To investigate patterns and factors associated with long-term initiation and follow-up of HIV, treatment and health outcomes among ALHIV, we established a matched social science cohort, linking survey data from Mzantsi Wakho participants—ALHIV in Eastern Cape, South Africa—to their National Health Laboratory Services (NHLS) laboratory tests routinely conducted in health facilities in South Africa. The aims of the Understanding Predictors of Lifelong Initiation and Follow-up Treatment for ALHIV (UPLIFT) cohort were to (1) supplement the previously abstracted laboratory data for participants from the ‘Mzantsi Wakho’ to include laboratory data from the NHLS; (2) characterise adolescents’ long-term HIV-related outcomes (retention on treatment, immunologic and virologic outcomes) and (3) identify what childhood and adolescent experiences, at multiple levels of their socioecological environment shape later health and HIV-related outcomes among ALHIV as they transition in adulthood.

## Cohort description

### Study setting

Participants in the UPLIFT cohort were enrolled from the larger ‘Mzantsi Wakho’ cohort study of ALHIV in Eastern Cape province, South Africa, who were followed up from 2014 to 2018 for three waves of self-reported interviews. The provincial HIV prevalence in nationally representative surveys was 25.2%, with 5–24-year olds, reporting the lowest rates of VL suppression (47% among females and 49% among males).^12 14 24^

### Study eligibility and data collection

Participants were eligible for UPLIFT if they were interviewed at least once during the ‘Mzantsi Wakho’ study (n=1563, figure 1) and provided consent for the use of their sociodemographic details to abstract their routine clinical or laboratory test records. Baseline recruitment into ‘Mzantsi Wakho’ was conducted from 2014 to 2015 with eligible participants identified from health records of all 52 clinics providing ART to at least more than five ALHIV in a large urban, peri-urban and rural health district of the province. Eligible Mzantsi Wakhp participants were traced back to their communities, and if consenting to study participation completed a survey on their well-being, which included SRH and HIV topics and abstraction of their routine health records. By 2018, a total of 1563 adolescents participated in the Mzantsi Wakho study. Of these, 1107 were known to be living with HIV while 456 were HIV negative or their status was unknown. The ‘Mzantsi Wakho’ study also recruited HIV-negative neighbouring or cohabiting peers of the participants living with HIV to prevent those living with HIV from being identified or stigmatised.^12 14^ Following completion of baseline interviews, clinical and laboratory records data including clinical appointments, medication history, WHO clinical staging and immunologic and virologic monitoring data were abstracted from participants’ clinic patient files in 2014–2015. Abstraction included review of hardcopy and electronic copies that were available at the health facilities, collecting data since each participant was diagnosed with HIV or started treatment. Four trained research assistants abstracted data using paper-based tools, which were then entered and merged with the main datasets, following procedures described elsewhere.^14^ During data linkages, all Mzantsi Wakho participants were considered eligible for UPLIFT.

**Figure 1:**
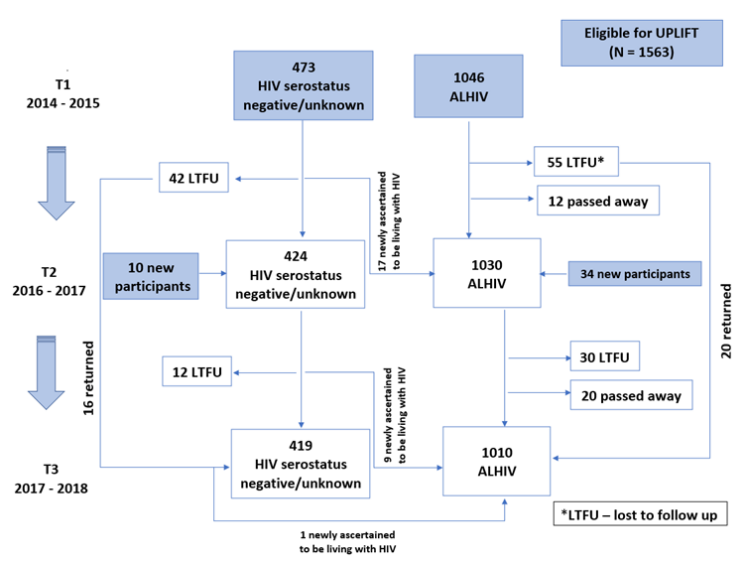
Flow diagram showing Mzantsi Wakho participants all of whom were eligible for inclusion into the UPLIFT cohort. *T1-3 represent the time of data collection with T1 as baseline. ALHIV, adolescent living with HIV; LFTU, lost to study follow-up; UPLIFT, Understanding Predictors of Lifelong Initiation and Follow-up Treatment for ALHIV.

### National Health Laboratory Services UPLIFT data linkage process

Following the consent of study participants and their caregivers in 2018, a partnership was established with the National Institute for Communicable Diseases (NICD), a division of the NHLS, to match the sociodemographic details of study participants with laboratory test records from the NICD data warehouse.

The NHLS provides nearly all laboratory-based diagnostic pathology services for South Africa’s public-sector health system, including all CD4 counts, and HIV VL tests conducted in public-sector facilities since the ART rollout in 2004. All HIV laboratory test results, among others, are uploaded to the NICD Data Warehouse. Data from the NICD data warehouse for matched participants were extracted in three rounds. The first round of data searches and extraction for ALHIV (UPLIFT cohort) from the ‘Mzantsi Wakho’ study was conducted between November 2020 and July 2021, with two follow-up rounds conducted between December 2021 to June 2022 and July to November 2023, respectively.

Participants’ personal sociodemographic details (names, surnames, sex, date of birth, clinic name and national identification numbers) were used to search the NICD data warehouse using iteratively refined searches. National identification numbers—a 13-digit unique identifier, assigned to South African citizens on registration at birth or when they become citizens, was collected from participants during interviews in 2018–2019 but was not available for all adolescents and was used as a supplementary matching detail. Sociodemographic details of potential matches, received from the NICD in Microsoft Excel (.xlsx) format, were further screened by a team of research assistants using an automated process. To ensure the accuracy of matched participant records, the R package *stringdist* was used to calculate pairwise string distances—measuring dissimilarities between text entries—and we also computed differences in date of birth records. Study participants were ascertained to be a match to an NHLS record, if all their details (name, surname and date of birth) were an exact match. In cases where names and surnames were exactly identical but not date of birth, we accepted date of birth records, which were exactly 1 day or 1 month off, or when the day and month of birth were reversed. In cases of discrepancies in names and surnames, researchers with knowledge of the South African language of participants in the Mzantsi Wakho cohort—IsiXhosa—assessed if these were locally acceptable variations of participant names. In addition, where study participants had a test result from the clinical and laboratory file data abstraction in 2014/2015,^14^ this was used as supporting evidence to confirm matches if the same results were identified in the NHLS laboratory records. Once personal identifiers were deemed a match, the corresponding NHLS test results were released in Microsoft Excel (.xlsx) format. Since NHLS test records are stored at a national level, the linkage of laboratory data to individual study participants was not dependent on participants receiving testing within the study area. This enables us to identify participant laboratory records even if they have transferred out of the study area to receive treatment.

The criteria for classifying participants as matches remained consistent between study rounds (table 1). However, to improve the success rate of the searches in round 2, we conducted three distinct searches using: (1) the list of eligible participants never matched; (2) list of eligible participants never matched but with a clinical or laboratory record from the previous clinic patient file abstraction and (3) all eligible participants. In round 3, the search was performed on the entire list of eligible participants to identify any NHLS test record that may have been missed.

**Table 1:** Summary of ALHIV matched between rounds 1–3 and criteria used to screen data obtained from the NICD data warehouse

	Matching criteria	Number of ALHIV matched*(N=1107)	Number of adolescents not living with HIV or HIV status unknown(N=456)
Round 1 (2021)	See footnote*	814 (73.5%)	46 (10.1%)
Round 2 (2022)	Same as round 1	850 (76.0%)	97 (21.2)
Round 3 (2023)	Same as round 1	852 (77.0%)	104 (22.8)

* *Exact match:* Name, surname and date of birth all exactly identical. *Potential match:* Name and surname exactly identical (or locally acceptable variations) plus date of birth 1 day of 1 month off, or day and month of birth reversed or date of birth difference<90 days. *No match:* name and surname not identical and not a locally acceptable variation and date of birth variation not within acceptable range.

ALHIV, adolescent living with HIV; NICD, National Institute for Communicable Diseases.

### Data management

During the first year of the study, protocols for data sharing, linking and storing were established to ensure accordance with the South African Protection of Personal Information act enacted in 2021.^25 26^ This included established secure server platforms, also used to transfer the NHLS data to the study team, with restricted access to four research team members who were responsible for matching records from NHLS to study participants. Research team members who were only involved in the subsequent analysis were not given access to any patient-identifying information. All non-matched sociodemographic data—data for individuals who were assessed not to have fulfilled the matching criteria for the UPLIFT cohort—received from NHLS were stored in a password-encrypted folder, to be erased or deleted permanently after finalising the linkage process. Data were received from NHLS in Microsoft Excel files and matched with the Mzantsi Wakho cohort data in R V.4.1.2, which was also used to analyse the data. Common variables used in describing the cohort included age, sex, mode of HIV acquisition—operationalised through an algorithm developed and tested by the team,^27^ residential area (urban/peri-urban or rural), orphanhood—defined using UNICEF criteria,^28^ and living with a biological caregiver based on the most recent participant interview, household poverty based on access to eight basic necessities used in prior research with children and adolescents in South Africa, and past-week adherence, using items adapted from existing tools and validated against VL data abstracted in 2014/2015.^29^ Full questionnaires are available here.

### UPLIFT participant characteristics

A total of 956 (61.2%) of the 1563 adolescents (852 of 1107 (77%) ALHIV and 104 of 456 (22.8%) not living with HIV or HIV status unknown) from the Mzantsi Wakho cohort were included in the UPLIFT cohort through at least one NHLS test result match (table 1). Just over half (57%) of the participants in the cohort were girls and 76% acquired HIV vertically (table 2). Further characteristics of all participants who are included in the UPLIFT study and those eligible for the study but had no matched test records are presented in table 2, online supplemental table S1. There were no significant differences between the sociodemographic characteristics of Mzantsi Wakho participants included in the UPLIFT cohort and those without matched NHLS test results. While we could not document reasons why existing Mzantsi Wakho participants were not matched with NHLS records, but prior research suggests that incorrect name spelling and record-keeping, and no national identifiers limit the matching and linkages in the data eco-system.^30 31^

**Table 2:** Characteristics of Mzantsi Wakho participants living with HIV (eligible for UPLIFT) and those matched and unmatched to any NHLS test record at their last interview in the ‘Mzantsi Wakho’ study

Characteristic	ALHIV eligible(N=1107)	UPLIFT(N=852)	Unmatched(N=255)	P value*
Age in years, mean (SD)	16.4 (3.09)	16.3 (2.99)	16.6 (3.41)	0.6
Sex, n (%)				
Boy	476 (43.0)	366 (43.0)	110 (43.1)	1.0
Girl	631 (57.0)	486 (57.0)	145 (56.9)	
Mode of HIV acquisition, n (%)				
Vertically acquired	823 (74.3)	647 (75.9)	176 (69.0)	0.5
Recently acquired	261 (23.5)	192 (22.5)	69 (27.1)	
Missing	23 (2.1)	13 (1.5)	10 (3.9)	
Residence area, n (%)				
Urban/peri-urban	844 (76.2)	641 (75.2)	203 (79.6)	0.6
Rural	263 (23.8)	211 (24.8)	52 (20.4)	
Orphanhood, n (%)	730 (65.9)	559 (65.6)	171 (67.1)	0.9
Biological caregiver, n (%)	467 (42.2)	367 (43.1)	100 (39.2)	0.7
Adolescent-reported poverty, n (%)	758 (68.5)	579 (68.0)	179 (70.2)	0.8
Self-reported past-week ART adherence, n (%)	804 (72.6)	628 (73.7)	176 (69.0)	0.6

Characteristics of the full sample (including those not living with HIV are in online supplemental table S1).

* P values for t-test (age) and $x^{2}$test for proportions. Comparisons are between those matched and unmatched.

ALHIV, adolescent living with HIV; ART, antiretroviral therapy; NHLS, National Health Laboratory Services; UPLIFT, Understanding Predictors of Lifelong Initiation and Follow-up Treatment for ALHIV.

### Summary of NHLS laboratory results

**Figure 2:**
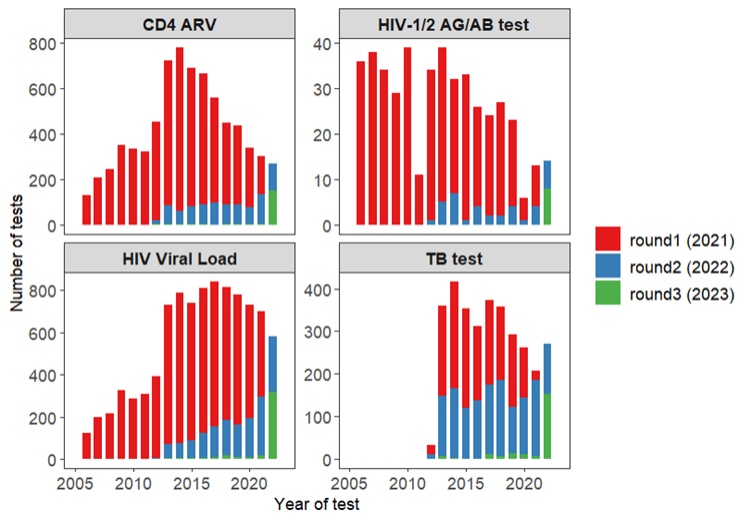
Total number of CD4 count (CD4 ARV), viral load, HIV rapid and tuberculosis (TB) test results extracted for matched participants over time.

A total of 32 886 individual laboratory test records, including 6801 CD4 counts and 8864 HIV VL test results, have been extracted for matched study participants. The results include tests conducted between 2004 and 2023, with the majority of test results from 2013 onwards. The distribution of extracted CD4 count, VL, HIV rapid test and tuberculosis test results over time are presented in figure 2, and a summary of the total number of laboratory test results extracted for matched study participants is presented in table 3. These tests—spanning a period from 2004 at the earliest matched result to most recent abstract date in 2023—will be analysed in future manuscripts to investigate a series of potential research topics introduced in the Future plans section.

**Table 3:** Laboratory tests extracted from the NICD data warehouse for matched UPLIFT participants (n=956)

Test category	Laboratory test type	Number of individual results (median number of results per matched participant and IQR (IQR))
HIV-related tests	CD4 count	6801 (7, IQR: 5–11)
	HIV RNA (viral load)	8864 (10, IQR: 7–14)
	Tuberculosis results (GeneXpert and microscopy)	2067 (3, IQR: 2–5)
	HIV ELISA (HIV-1/2 AG/AB): screening and confirmatory	251 (2, IQR: 1–2)
Other tests	Anatomical pathology	214 (1, IQR: 1–2)
	Chemical pathology	8745 (8, IQR: 6–12)
	Haematology	5219 (4, IQR: 3–7)
	Microbiology	725 (1, IQR: 1–2)

NICD, National Institute for Communicable Diseases; UPLIFT, Understanding Predictors of Lifelong Initiation and Follow-up Treatment for adolescent living with HIV.

## Findings to date

Using merged social science data and routine data from UPLIFT, we assessed the validity of self-reported ART adherence measures in predicting elevated VL defined as a VL ≥1000 copies/mL. In this assessment, self-reported adherence measures reported over the three study rounds conducted between 2014 and 2018 were found to be significantly associated with an elevated VL in this cohort of ALHIV.^32^ Self-reported measures most likely to predict an elevated VL were missed doses in the past 3 days, in the past week, and any missed clinic appointment in the past year. A combination of these three measures significantly improved their sensitivity in predicting an elevated VL among UPLIFT participants.^32^

The second analyses group-based multitrajectory modelling to characterise long-term ART adherence trajectories among ALHIV based on social science self-reported data. This analysis found four distinct long-term adherence patterns—consistent adherence, low start and increasing adherence, good start and gradually decreasing, and low and decreasing ART adherence—among ALHIV on ART.^33^ Around half (49.8%) of adolescents were found to have ‘consistent adherence’ with 20.8% having initially poor but increasing adherence over time. Adherence was ‘gradually decreasing’ in 23.5% and ‘low and decreasing’ among 5.9% of ALHIV. An assessment of the association between durable viral suppression, defined as two consecutive VL measurements <1000 copies/mL during the study period from UPLIFT and adherence trajectories found that the odds of durable viral suppression were highest in the ‘consistent adherence’ group compared with the other groups.^33^

VL data from the UPLIFT study have also been used to support evidence of a negative association between exposure to intimate partner violence or sexual abuse and self-reported ART adherence and VL suppression (VL <50 copies/mL).^34^ VL results were used to assess constructs from the WHO HEADSS and HEADSS+ (Home, Education/employment, peer group Activities, Drugs, Sexuality and Suicide/depression) checklists associated with non-adherence.^35^ The constructs most predictive of non-adherence among ALHIV were exposure to violence, depression, medication side effects and low social support.^35^

Additional research using this linked cohort found that adolescents who experienced food insecurity, excessive substance use, inequitable partnerships, early parenthood and with recently acquired HIV were more likely to report secondary HIV exposure risk (viremia and unprotected sex).^36^ Among adolescent girls and young women living with HIV, self-reported access to safe and affordable facilities, defined as reporting ability to afford to get to the doctor, clinic or hospital, and feeling safe at the clinic/hospital in the past year and self-reporting access to kind and respectful healthcare providers is associated with higher predicted probability of ART adherence, viral suppression, no TB symptoms and uninterrupted ART treatment.^37^

## Future plans

The UPLIFT cohort is a well-characterised cohort of ALHIV initiated on ART in the Eastern Cape, South Africa with linked laboratory data from the National Health Laboratory Service in South Africa for the majority of adolescents in the cohort. These data provide a unique opportunity for monitoring trends in future immunologic, virologic and other laboratory indicators among ALHIV on treatment. The availability of linked questionnaire interview data on medication-related experiences, SRH practices, mental health well-being, social and structural measures will enable us to understand how these characteristics influence future treatment outcomes among this young population living with HIV. We plan to investigate how individual, familial, community and healthcare experiences in childhood, early and mid-adolescence shape long-term health outcomes, including HIV treatment outcomes, among this priority population. Since the COVID-19 pandemic happened during the period of matched data, we will also investigate the potential effect of the COVID-19 pandemic on adolescent HIV treatment outcomes.

## Collaboration

The UPLIFT cohort is an existing collaboration between the Universities of Cape Town, Oxford, and the NICD, NHLS in South Africa. Analyses of the UPLIFT data have been the backbone for postgraduate training for four doctoral (Haghighat, Zhou, Tolmay and Edun), two masters (He and Leon) and several postdoctoral students. De-identified data are available for non-profit use, particularly postgraduate training of under-represented early career researchers, following data access protocols available at http://www.mzantsiwakho.org.za/.

## Patient and public involvement

Adolescents informed the creation of the UPLIFT data through a Teen Advisory Group (TAG) arts-based participatory research. TAG, a group of (15–25) AIDS-affected adolescents—some from the parent study—was formed to engage adolescents and young people as cocreators of social science research and to develop adolescent-informed policy and programming recommendations. TAG provided critical input to study designs with reflections on what to include in questionnaires and methodologies to ethically and sensitively recruit and retain study participants.^38 39^ In one of the many TAG meetings, convened in 2019, adolescents and young people spoke about the need to be seen beyond the clinics. The UPLIFT data are response to these recommendations, following iterative engagements with adolescents. The dissemination networks for the study’s findings were informed by a community audit and stakeholder mapping, which has enhanced the reach of the study’s impact activities and ensured findings are disseminated to stakeholders with major influence in adolescents’ HIV/AIDS policy and programming.

## Supplementary material

10.1136/bmjopen-2024-092909 online supplemental file 1

## Data Availability

Data are available upon reasonable request.
