# Supplementary material for: Understanding Predictors of Lifelong Initiation and Follow-up Treatment for adolescents and youth living with HIV (UPLIFT): an integrated prospective cohort in Eastern Cape, South Africa
Source: BMJ Open. 2025 Jul 8;15(7):e092909. doi: 10.1136/bmjopen-2024-092909 (PMC12243594; doi:10.1136/bmjopen-2024-092909)
Supplement: online supplemental file 1 [file bmjopen-15-7-s001.docx]

**Supplementary materials**

**Table S1.** Characteristics of all Mzantsi Wakho participants (eligible for UPLIFT) and those matched and unmatched to any NHLS test record at their last interview in the ‘Mzantsi Wakho’ study

| **Characteristic** | **All Mzantsi Wakho participants (eligible)**  **(N=1563)** | **Participants matched**  **to any NHLS test**  **(N = 956)** | **Unmatched participants**  **(N = 607)** | ***p-value** |
| --- | --- | --- | --- | --- |
| Age in years, mean (SD) | 16.5 (3.10) | 16.5 (3.02) | 16.5 (3.21) | 0.70 |
| **Sex, n (%)** |  |  |  |  |
| Boy | 657 (42.0) | 386 (40.4) | 271 (44.6) | 0.10 |
| Girl | 906 (58.0) | 570 (59.6) | 336 (55.4) |  |
| **Dwelling type, n (%)** |  |  |  |  |
| Urban | 1182 (75.6) | 702 (73.4) | 480 (79.1) | 0.01 |
| Rural | 381 (24.3) | 254 (26.6) | 127 (20.9) |  |
| **Biological Caregiver, n (%)** | 730 (46.7) | 435 (45.5) | 295 (48.6) | 0.30 |
| **Adolescent-reported poverty, n (%)** | 1051 (67.2) | 655 (68.5) | 396 (65.2) | 0.20 |
