# Supplementary material for: Understanding Predictors of Lifelong Initiation and Follow-up Treatment for adolescents and youth living with HIV (UPLIFT): an integrated prospective cohort in Eastern Cape, South Africa
Source: BMJ Open. 2025 Jul 8;15(7):e092909. doi: 10.1136/bmjopen-2024-092909 (PMC12243594; doi:10.1136/bmjopen-2024-092909)
Supplement: Supplementary file 2 [file bmjopen-2024-092909.reviewer_comments.pdf]

PEER REVIEW HISTORY

BMJ Open publishes all reviews undertaken for accepted manuscripts. Reviewers are asked to complete a checklist review form and are provided with free text boxes to elaborate on their assessment. These free text comments are reproduced below.

ARTICLE DETAILS

Title (Provisional)

Cohort profile: Understanding Predictors of Lifelong Initiation and Follow-up Treatment for Adolescents and Youth living with HIV (UPLIFT): an integrated prospective cohort in Eastern Cape, South Africa

Authors

Toska, Elona; Edun, Olanrewaju; Zhou, Siyanai; Leon, Zea; Langwenya, Nontokozo; Jochim, Janina; Tolmay, Janke; Sherman, Gayle; Cluver, Lucie Dale

---

VERSION 1 - REVIEW

**Reviewer**	**1**
**Name**	**Hatzold, Karin**
**Affiliation**	**Population Services International, HIV and TB**
**Date**	**22-Sep-2024**
**COI**	**None**

The paper has significant weaknesses and does not adhere to the standard structure of a scientific manuscript. It lacks a proper discussion section, a clear results section, and conclusions that are based on the presented data. The abstract is also poorly structured and does not follow the typical format of introduction/background, methodology, results, and conclusion.

The methodology used to arrive at the results is unclear, particularly in regard to the associations or predictors of adherence to treatment and treatment outcomes. This appears to be a cross-sectional description of factors related to laboratory results (e.g., viral load, CD4 cell count), but the issue of causality is not adequately addressed. Moreover, the results are not presented in any table, making it difficult to evaluate the findings.

Additionally, the manuscript does not meet the standard of written English required for publication.

**** The reviewer provided a marked copy with additional comments. Please contact the publisher for full details. ****

Reviewer 2

Name Naidoo, Inbarani

Affiliation Human Sciences Research Council, Public Health Societies and Belonging

Date 05-Feb-2025

COI I have collaborated with some co-authors on another project.

Dear Editor

Thank you for the opportunity to review the paper entitled “Cohort profile: Understanding Predictors of Lifelong Initiation and Follow-up Treatment for Adolescents and Youth living with HIV (UPLIFT): a prospective cohort of matched social science and routine laboratory data in Eastern Cape, South Africa”

The authors describe linking several laboratory measures (HIV testing, ART uptake and viral load suppression, SRH services, mental health, locality type) with cohort data for adolescents and young people aged 10-19 years living with HIV in Eastern Cape within the Mzantsi Wakho study. This is a novel approach to mining available data on adolescents living with HIV and their HIV negative counterparts for the Eastern Cape province in South Africa. The authors refer to previously published papers where they analysed the linked cohort dataset for self-reported ART adherence and viral load, and ART adherence trajectories. The authors further cite a previously published paper looking at socio-behavioural factors including access to healthcare, their experiences within the healthcare setting and HIV risk exposure.

Please find specific suggestions for each section in the below table.

Thank you for the opportunity to review.

Paper title	Suggest shortening the title
Page 4	The authors mention the impact of COVID-19 will be assessment on HIV treatment outcomes. Hence will HIV test results be linked to COVID test results? Also will COVID vaccination be included?
Page 7<br>Cohort description	Suggest specifying the software used to link the datasets, and to calculate the t-test and $x2$ test for proportions (matched vs unmatched) as described in Tables 2 and S1.<br>What data format was the NHLS data accessed in and how were the common fields linked? Was it an automatic or manual merge of data?
Page 8<br>Rows 24-25	Suggest elaborating on how the data abstraction was conducted? Were the clinical and laboratory measures captured on paper or a digital system?
Page 9	The authors state that national identification numbers were used to match Mzantsi Wakho participants in the NHLS database. Suggest describing the format national identification number e.g. 13 digit identity number, what about adolescents who did not have an identification number?
Page 10<br>Rows 24-34<br>Table 1	The authors state "Having a laboratory test result identical to that previously obtained from the clinical and laboratory data abstraction in<br>2014/2015 was used as supporting evidence to confirm matches."<br>Suggest clarifying this statement to match dates from the different data abstractions (data abstraction conducted in 2014/2015 vs NHLS data mining).<br>Would it be possible for an adolescent to have a HIV negative test result at the time of the clinical and laboratory data abstraction in 2014/2015 and then test positive at the time of presenting at the health facility within the 2014/2015 reporting period? If this is possible, how were discordance results handled in the matching process?
Page 10<br>Row 57	Suggest writing Protection of Personal Information Act in full then abbreviate to POPI
Page 11<br>Row 23	Please clarify which "68% acquired HIV vertically." The proportion of adolescents who acquired HIV vertically is listed in Table 2 and not listed in S1
Page 11<br>Table 2	Table 2 lists "dwelling type" as urban/rural. Suggest specifying how urban/rural were defined in relation to dwelling type rather than locality type. Page 8 row 9 mentions three locality types namely urban/peri-urban/rural according to Eastern Cape health districts.
Page 12<br>Table 2	Suggest specifying the type of orphanhood (i.e. maternal or paternal or double orphan)

Page 12<br>Figure 2	The numbers of tests retrieved for CD4 count, viral load, HIV rapid test and tuberculosis over time are reported in Figure 2. Suggest elaborting why these testing numbers are noteworthy within the linked cohort study. The “year of test” axix appears to start in 2006, which is not aligned with the Mzantsi Wakho cohort study start time of 2014.

**Reviewer**	**3**
**Name**	**Lyatuu, Goodluck**
**Affiliation**	**Management and Development for Health, Programs**
**Date**	**27-May-2025**
**COI**	**None**

This manuscript describes a profile of a cohort of adolescents living with HIV (ALHIV) receiving HIV care in Eastern Cape, South Africa, linking clinical and laboratory data with their self-reported social experiences obtained from interviews with the ALHIV. The manuscript and the cohort address an important gap in research and provide a unique and valuable opportunity to study the interaction between social characteristics and clinical/ laboratory parameters of ALHIV and how they influence HIV treatment outcomes, particularly for this sub-group which is lagging in the efforts to end AIDS as a public health threat in sub-Saharan Africa and the world at large. Detailed comments are provided in the attached document

Title: Cohort profile: Understanding Predictors of Lifelong Initiation and Follow-up Treatment for Adolescents and Youth living with HIV (UPLIFT): a prospective cohort of matched social science and routine laboratory data in Eastern Cape, South Africa

Summary: This manuscript describes a profile of a cohort of adolescents living with HIV (ALHIV) receiving HIV care in Eastern Cape, South Africa. The cohort links clinical and laboratory data on the HIV care of the ALHIV with their self-reported social experiences obtained from interviews with the ALHIV. This manuscript and the cohort address an important gap in literature, and provide a unique and valuable opportunity to study the interaction between social characteristics and clinical/ laboratory parameters of ALHIV and how they influence HIV treatment outcomes, particularly for this sub-group which is lagging in the efforts to end AIDS as a public health threat in SSA and globally

Comments
Abstract

- The abstract is too long. The Purpose and Future plans can be substantially shortened to make them summarized and succinct
- Under “Participants” also describe how the participants were identified and recruited. Also does the cohort’s target population include “adolescents not living with HIV”. This is not clear/contradicting between sections (i.e. Purpose vs

Participants) and under Study Settings it has been described as a measure to mitigate stigma (as opposed to a target population)?
- Revise the “Findings to date” to provide some descriptive data (i.e. on the self-reported adherence, viral suppression…) before making associations (lines 51-60). The first part of the “Findings to date” section (lines 43-48) can also be shortened i.e. information on the absolute number of records that matched is less informative.

Introduction
- The introduction is well written with a motivating and persuasive rationale

Cohort description
- Expand the description of Study setting to include key statistics about the setting i.e. on HIV overall and among adolescents. Also, will be useful to know the standard of care at the setting i.e. when is CD4/ viral load/ TB/ other testing offered, how frequent etc. Some of the content on participants recruitment (i.e. lines 3-18) should be moved under Study eligibility/Participants selection. Also lines 19-25 should be reported under data collection.
- Under Data collection/extraction, also specify what data was/ is collected/extracted and how it was measured. Ideally all data reported under results should be described in methods specifying how it was obtained and analysed.
- Table 1 can be improved by moving information on criteria to a table footnote.
- Under the “UPLIFT participants characteristics”, provide some insights as to why some records did not match
- In Table 3, reporting absolute number of results extracted alone, provides limited information to assess against what is expected. You may reorganize into number of results per study participant/ provide some benchmark.
- **Future plans and collaboration** sections are well described.

VERSION 1 - AUTHOR RESPONSE

Reviewer feedback and author responses

Comment	*Response*
**Reviewer 2**	
Paper title: Suggest shortening the title	Thank you for this suggestion, we have edited the title: Cohort profile: Understanding Predictors of Lifelong Initiation and Follow-up Treatment for Adolescents and Youth living with HIV (UPLIFT): an integrated prospective cohort in Eastern Cape, South Africa

Page 4: The authors mention the impact of COVID-19 will be assessment on HIV treatment outcomes. Hence will HIV test results be linked to COVID test results? Also will COVID vaccination be included?	Thank you for this great question. We did not collect data from all participants since 2021, and while the NHLS data includes COVID-19 tests and results, these were only available for a small proportion of the participants (n=101). Therefore ,we cannot include individual-level COVID19 results – and we have clarified this in the revised manuscript. However, we have started and completed analyses using different periods of the COVID-19 pandemic (before, during and after), using the cohort data, and have noted the possibility of analysing who had linked COVID-19 tests.
Page 7: Cohort description<br>Suggest specifying the software used to link the datasets, and to calculate the t-test and $x2$ test for proportions (matched vs unmatched) as described in Tables 2 and S1.<br><br>What data format was the NHLS data accessed in and how were the common fields linked? Was it an automatic or manual merge of data?	We have added the software used for merging, data checks, and analyses (R version 4.1.2) in the manuscript. Thank you for suggesting this detail.<br><br>The NHLS data were provided as password-protected Microsoft Excel files (.xlsx format) containing all the potential matches. Common fields were linked through an automated process that utilized the R package *stringdist* to calculate pairwise string distances, enabling measurement of dissimilarities between text entries. Differences in date of birth records were also computed using R version 4.1.2. These procedures are now described in the methods section, alongside the criteria used to
	determine whether study participants were considered a match, previously outlined.

Page 8 (Rows 24-25): Suggest elaborating on how the data abstraction was conducted? Were the clinical and laboratory measures captured on paper or a digital system?	Thank you for suggesting this improvement. Since we have published details of it in prior manuscripts, we have edited this profile to include references to them and added some brief detail as suggested by the reviewer.
Page 9: The authors state that national identification numbers were used to match Mzantsi Wakho participants in the NHLS database. Suggest describing the format national identification number e.g. 13 digit identity number, what about adolescents who did not have an identification number?	Thank you for picking this up. We have edited this section to clarify what the national identity number is, how it was collected and its use as a supplementary matching detail.
Page 10 (Rows 24-34) Table 1: The authors state "Having a laboratory test result identical to that previously obtained from the clinical and laboratory data abstraction in 2014/2015 was used as supporting evidence to confirm matches."<br><br>Suggest clarifying this statement to match dates from the different data abstractions (data abstraction conducted in 2014/2015 vs NHLS data mining).<br><br>Would it be possible for an adolescent to have a HIV negative test result at the time of the clinical and laboratory data abstraction in 2014/2015 and then test positive at the time of presenting at the health facility within the 2014/2015 reporting period? If this is possible, how were discordance results handled in the matching process?	Thank you for identifying this lack of clarity. We have edited Table 1 and clarified the methods section, where we outline what data points were used for matching.<br><br>The data abstracted in 2014/2015 covered the period from participants diagnosis/ treatment start date until the date of extraction. When a match was noted within NHLS data, including an exact match of abstracted data from Mzantsi Wakho participants and NHLS data, this confirmed the match, even when other data points may not be perfect matches. For any NHLS data following the data abstraction date, other details were used for matching the NHLS data and Mzantsi Wakho participant data, as described in the manuscript. We have clarified this process and timelines in the manuscript.
Page 10 (Row 57): Suggest writing Protection Personal Information Act in full then abbreviate to POPI	Thank you – we have done this the first time POPIA is mentioned.
Page 11 (Row 23): Please clarify which "68% acquired HIV vertically." The proportion of adolescents who acquired HIV vertically is listed in Table 2 and not listed in S1.	Thank you so much for pointing this out. We have corrected the statistic for mode of HIV acquisition and the reference to Table 2 (rather than S1).

	We did not include the mode of HIV acquisition in S1 because the table includes all participants, even participants not living with HIV.
Page 11 (Table 2): Table 2 lists “dwelling type” as urban/rural. Suggest specifying how urban/rural were defined in relation to dwelling type rather than locality type. Page 8 row 9 mentions three locality types namely urban/peri-urban/rural according to Eastern Cape health districts.	Thank you for identifying this inconsistency. We have edited Table 2, and clarified how the variable was defined in page 11.
Page 12 (Table 2): Suggest specifying the type of orphanhood (i.e. maternal or paternal or double orphan)	Thank you for suggesting this clarification. We have added the details for how each variable reported in the Tables and manuscript were measured and operationalised with references as relevant.
Page 12 (Figure 2): The numbers of tests retrieved for CD4 count, viral load, HIV rapid test and tuberculosis over time are reported in Figure 2. Suggest elaborating why these testing numbers are noteworthy within the linked cohort study. The “year of test” axis appears to start in 2006, which is not aligned with the Mzantsi Wakho cohort study start time of 2014.	This is a really important point. We have clarified in page 12 and also in other parts of the manuscript what the period of abstracted data is for all tests. We have added some clarifying notes to Figure 2.
**Reviewer 3**	
Comment	Response
The abstract is too long. The Purpose and Future plans can be substantially shortened to make them summarized and succinct	Thank you for your feedback. We have edited the abstract to make it more concise.
Abstract: Under “Participants” also describe how the participants were identified and recruited. Also does the cohort’s target population include “adolescents not living with HIV”. This is not clear/contradicting between sections (i.e. Purpose vs Participants) and under Study Settings it has been described as a measure to mitigate stigma (as opposed to a target population)?	Thank you for this suggestion. We have explained this briefly in the abstract but also improved the methods of the paper.

Abstract: Revise the "Findings to date" to provide some descriptive data (i.e. on the self-reported adherence, viral suppression…) before making associations (lines 51-60). The first part of the "Findings to date" section (lines 43-48) can also be	Thank you for this suggestion – we have added some findings.
shortened i.e. information on the absolute number of records that matched is less informative.	
Introduction: The introduction is well written with a motivating and persuasive rationale	Thank you for this supportive feedback.
Expand the description of Study setting to include key statistics about the setting i.e. on HIV overall and among adolescents. Also, will be useful to know the standard of care at the setting i.e. when is CD4/ viral load/ TB/ other testing offered, how frequent etc.	We have added this information to the revised study setting, thank you for suggesting it.
Some of the content on participants recruitment (i.e. lines 3-18) should be moved under Study eligibility/Participants selection.	Thank you for suggesting this. We have edited these sections.
Also lines 19-25 should be reported under data collection.	We have addressed this feedback in the edited manuscript. Thank you.
Under Data collection/extraction, also specify what data was/ is collected/ extracted and how it was measured. Ideally all data reported under results should be described in methods specifying how it was obtained and analysed.	Thank you for this suggestion. We have added this information and the relevant references.
Table 1 can be improved by moving information on criteria to a table footnote.	Thank you for this suggestion, which was very helpful. We hope the edited Table 1 is clearer.
Under the "UPLIFT participants characteristics", provide some insights as to why some records did not match	Thank you for this suggestion. We have added some potential reasons but also references to other studies that have looked into this.
In Table 3, reporting absolute number of results extracted alone, provides limited information to assess against what is expected. You may reorganize into number of results per study participant/ provide some benchmark.	Thank you for this suggestion. We have added the median (IQR) for the number of tests matched per individual participant.
**Future plans and collaboration** sections are well described	Thank you so much.
